# Tobacco as an Antizymasic

**Published:** 1885-07

**Authors:** 


### Tobacco as an Antizymasic.

M. Pecholier in the *Montpelier Medical* considers that the use of tobacco preserves one from an infinity of contagious diseases. He thinks that at typhoid fever is due to a ferment, the pollulation and life of which in the organism is the initial cause of the disease, tobacco is the most powerful destructive agent, and that its action is due to nicotine ; he declares that a number of smokers have been protected from epidemic influences through tobacco. Perhaps it is for this reason that Willis recommends the use of tobacco in armies, as a preservative against certain epidemic diseases.

